# The impact of remote monitoring on clinical outcomes in defibrillator patients during the COVID-19 pandemic: An interrupted time series analysis

**DOI:** 10.1016/j.hroo.2026.06.008

**Published:** 2026-06-19

**Authors:** Sandeep Chand, Barbara Torres, David M. Vickers, Tara A. Whitten, Andrew Mardell, Glen Sumner

**Affiliations:** 1 Community Health Sciences, Cumming School of Medicine, University of Calgary, Alberta, Canada; 2 Alberta Health Services, Alberta, Canada; 3 Mozell Core Analysis Lab, Libin Cardiovascular Institute, Cumming School of Medicine, University of Calgary, Alberta, Canada; 4 Provincial Research Data Services, Alberta Health Services, Alberta, Canada; 5 Data and Research Services, Alberta SPOR SUPPORT Unit, Alberta, Canada; 6 Department of Cardiology, Libin Cardiovascular Institute, University of Calgary, Alberta, Canada

**Keywords:** Cardiovascular implantable electronic devices, COVID-19, Implantable cardioverter-defibrillators, Interrupted time series, Remote monitoring

## Abstract

**Background:**

Remote monitoring (RM) has become the standard of care for managing patients with defibrillators. Early in the coronavirus disease 2019 (COVID-19) pandemic, there were abrupt constraints to in-person device clinic access. The effect on overall device clinic access and clinical outcomes in the defibrillator population with and without RM during this time frame is uncertain.

**Objective:**

This study aimed to examine the effect of the early period of the COVID-19 pandemic on health care access and clinical outcomes in patients with a defibrillator with either an RM or non-RM model of care.

**Methods:**

Administrative data from the province of Alberta, Canada, were evaluated between March 17, 2018, and July 17, 2021, to observe and compare secular and pandemic-associated trends in overall visits, emergency department visits, cardiovascular hospitalizations, and all-cause mortality in the period before (prepandemic) and after March 16, 2020 (intrapandemic), between RM and non-RM patients with defibrillators. We applied interrupted time series analysis, and the significance for statistical tests was set at 5%.

**Results:**

Of 4604 adults with defibrillators, 80% had RM. The onset of the pandemic was associated with an immediate increase in virtual care visits that was much greater in the RM group. Clinical outcome trends in both groups were essentially unchanged although we observed a 2-fold higher baseline mortality in the non-RM group that minimally declined after the pandemic.

**Conclusion:**

During the early period of the COVID-19 pandemic, RM patients had significantly improved access to virtual device clinic care compared with non-RM patients. However, clinical outcome trends were essentially unchanged.

**Key Findings**

▪ The onset of the pandemic demonstrated improved cardiovascular implantable electronic device access to care in remote monitoring (RM) patients, driven by an increased number of virtual care visits.
▪ RM and non-RM patients experienced similar pandemic-related disruption to access to in-person care.
▪ The pandemic had minimal effect on trends for cardiovascular hospitalizations, emergency department visits, or mortality for both the RM and non-RM groups.

## Introduction

Severe acute respiratory syndrome coronavirus 2 infection was declared a world pandemic by the World Health Organization on March 11, 2020.^1^ The coronavirus disease 2019 (COVID-19) pandemic triggered a significant shift in health care delivery, prompting policy makers and health care providers to rapidly transform how patients are monitored and treated. Among the various adaptations in health care, an amplification of virtual care visits and remote monitoring (RM) have emerged as pivotal strategies, particularly for patients with cardiovascular implantable electronic devices (CIEDs).^2^

RM allows for the timely transmission of CIED-stored diagnostic data to a central server and analysis of these data by a trained nurse clinician. If warranted, the nurse clinician may pass on this information to a physician who may intervene, dependent upon the findings. There is growing evidence that communication of the RM data to a heart rhythm specialist physician may improve quality of care.^3^ Furthermore, prompt detection of arrhythmia and of heart failure deterioration may decrease emergency department (ED) visits, hospital admissions, and hospital length of stay.^4^^,^^5^ In practice, there are now selected CIED clinics that have adopted an “alert-based” or RM-only care with CIED clinic visits reserved only for use on an urgent, ad hoc basis.^6^ Multiple guidelines have recommended the wide adoption of an RM strategy for patients with CIEDs.^7^^,^^8^ Although there is evidence that RM helps to reduce mortality and health care resource utilization, it is unclear whether this applies when access to in-person care is constrained, as occurred during the early phase of the COVID-19 pandemic.

We used routinely collected administrative data from device clinics in Alberta, Canada, to conduct an interrupted time series (ITS) analysis that assessed the effect of the early period of the COVID-19 pandemic on health care utilization and cardiovascular (CV) outcomes in patients with CIEDs. We focused our analysis on the population of people with defibrillators and compared those with and without RM.

## Objective

This study aimed to examine the effect of the COVID-19 pandemic on health care utilization and CV outcomes in patients with a defibrillator with or without an RM model of care.

## Methods

In this retrospective cohort study with an ITS analysis, we used aggregated and anonymized administrative outcome data maintained by the Alberta Health Services. The data provided comprehensive, cumulative, and longitudinal data for all known defibrillator patients in Alberta, Canada. This jurisdiction is a publicly financed, universal access health system with eligible residents enrolled in the provincial health plan. Outcomes were ascertained through linkage of defibrillator patients’ deidentified personal health numbers to the outcome databases using the International Classification of Diseases, 10th Revision (Supplemental Table 1).^9^ Because Alberta’s provincial cabinet declared a provincial public health emergency on March 17, 2020, we defined the preintervention study period as March 17, 2018, to March 16, 2020, with the intervention to be a specific 16-month time period of the COVID-19 pandemic in Alberta (intrapandemic) from March 17, 2020, to July 17, 2021.^10^ The research reported in this article adheres to the Declaration of Helsinki. This study was approved by the University of Calgary’s Conjoint Health Research Ethics Board with waiver of informed consent (REB21-0568).

### Setting

Implantable cardioverter-defibrillator (ICD) RM involves scheduled transmissions every 3–6 months, supplemented by automatic alerts for arrhythmias, shocks, device or lead abnormalities, and patient-initiated transmissions for symptoms.^11^ In Alberta, RM is integrated with routine in-clinic follow-up after initial device implantation, with device clinic staff reviewing transmissions and escalating clinically significant findings to electrophysiologists when required. Enrollment in RM remains optional for patients and is provided without direct cost.^11^^,^^12^ Several factors may influence RM participation, including patient preference for in-person clinic visits, desire for direct interaction with health care providers, limited comfort with technology among elderly patients, reduced engagement in self-management, socioeconomic and rural barriers, and variability in support for different proprietary RM platforms across Alberta clinics.^12^

### Study population

We included defibrillator patients aged 18 years and older in Alberta, Canada, who accessed CIED clinics between March 17, 2018, and July 17, 2021. We divided defibrillator patients into the RM and non-RM groups. A province-wide proprietary device clinic database, Paceart™ (Medtronic Corporation, Dublin, Ireland), was used to identify patients with or without RM status.^13^

Patients were classified as part of the RM cohort if they had ≤2 nonremote encounters, followed by remote or nonremote encounters for the remainder of the study period. The initial ≤2 nonremote encounters typically represented device implantation or generator replacement procedures. Patients who had only nonremote encounters throughout the study period were defined as nonremote. Patients not meeting the abovementioned criteria (eg, patients switching to RM or non-RM) were excluded from the analysis. We linked Paceart data to the discharge abstract database, the national ambulatory care reporting system, and vital statistics to assess baseline characteristics and outcomes. Comorbidities were identified using the International Classification of Diseases, 10th Revision, codes (Supplemental Table 1).^9^

### Outcomes

We compared trends in overall clinic visits (in-person visits and virtual consultations), ED visits, CV hospitalizations, and all-cause mortality in patients with defibrillators with and without RM. The exposure was the initial 16-month period of the pandemic as defined earlier. Thus, after 3 years, outcome data were determined with prepandemic secular trend and then analyzed during the pandemic for defibrillator patients with and without RM.

We assessed the volume of overall visits by combining in-person visits and virtual consultations. We defined virtual visits as any interaction between patients and CIED clinic members (nurse clinicians or physicians), including alert-based follow-ups or scheduled device transmissions, that was not conducted in person. ED visits were visits that resulted in discharge from the ED owing to heart failure, arrhythmias, stroke, or myocardial infarction that involved physician care in an emergency room or urgent care setting. These visits were identified using the national ambulatory care reporting system database. CV hospitalizations included in-hospital admissions for heart failure, arrhythmias, stroke, or myocardial infarction using the discharge abstract database. All-cause mortality was identified using the vital statistics database.

### Statistical analysis plan

χ^2^ or Fisher’s exact test was used as descriptive statistics of categorical variables between RM and non-RM. The dichotomous outcomes were measured as a proportion of the total number of ICD patients with RM vs non-RM in monthly intervals between March 17, 2018, and July 17, 2021. The denominator for each outcome was defined as the total number of active patients with a defibrillator device and with either RM or non-RM within a given month throughout the study period. Each outcome was plotted on a separate graph based on the best fit of data. To describe the trends in our outcome variables over time among RM and non-RM patients, we used 3 different regression models: linear regression model, ITS model, and segmented regression model (Supplemental Equation). We included ITS and segmented regression models because changes in intercept and slope from March 2018 to July 2021 were used to test hypotheses about the influence of the responses to COVID-19 on the outcomes listed earlier. The model with the lowest Akaike information criterion was deemed the best fit to the available data and reported in our study. We also evaluated serial correlation in model residuals up to order 8 using the Breusch-Godfrey test (Supplemental Table 2). To assess potential seasonality, we included monthly dummy variables in the models. If the inclusion of these variables altered the trend coefficient by more than 20%, seasonality was considered a time-varying confounder requiring adjustment. Outcome comparisons between RM and non-RM were described as differences with 95% confidence intervals (CIs) using appropriate statistical techniques for discrete variables. We used R statistical software (version 4.2.3) for analysis.

## Results

### Cohort characteristics

We identified a total of 4604 new adults with a defibrillator between March 17, 2018, and July 17, 2021, in Alberta. Approximately 80% patients were identified as having RM (Table 1). A significant proportion of patients were identified from the Calgary zone (58% non-RM vs 47% RM), followed by the Edmonton zone (45% non-RM vs 32% RM), whereas less than 5% of patients from the central and north zones were identified in both cohorts. The proportion of patients with cardiac resynchronization therapy was 37% in the non-RM group and 26% in the RM group (*P* < .001). The median age in the non-RM cohort was 66 years (interquartile range 18–93) and was statistically significant (*P* < .001) between 2 groups. There were 77% males in the non-RM and 78% in the RM groups (*P* = .6) (Table 1). The patients with heart failure (31% vs 19%), atrial fibrillation (16% vs 13%), and diabetes (26% vs 19%) were significantly higher in the non-RM group (*P* < .005) (Table 1).

**Table 1:** Baseline population characteristics for patients with ICD

Characteristic	Nonremote (n = 943)	Remote (n = 3661)	*P* value
CRT			<.0001∗
CRT	352 (37)	970 (26)	
Non-CRT	591 (63)	2691 (74)	
Age	66 (18–93)	67 (18–94)	.048∗
Sex			.6
Female	211 (23)	793 (22)	
Male	725 (77)	2857 (78)	
Zones			.0001∗
Calgary	439 (47)	2108 (58)	
Central	13 (1)	161 (4)	
Edmonton	424 (45)	1171 (32)	
North	1 (0)	2 (0)	
South	66 (7)	219 (6)	
Comorbidities			
Coronary and peripheral vascular disease	153 (16)	476 (13)	.010∗
Ischemic cardiomyopathy	198 (21)	697 (19)	.2
Nonischemic cardiomyopathy	83 (9)	296 (8)	.5
Heart failure	291 (31)	691 (19)	<.0001∗
Stroke	22 (2)	55 (2)	.076
Atrial fibrillation	150 (16)	460 (13)	.007∗
Ventricular tachycardia	19 (2)	65 (2)	.6
Ventricular arrhythmia	113 (12)	450 (12)	.8
Cardiac arrest	49 (5)	153 (4)	.2
Hypertension	256 (27)	888 (24)	.067
Diabetes	246 (26)	711 (19)	<.0001∗
Dyslipidemia	50 (5)	184 (5)	.7
Renal diseases	42 (4)	127 (3)	.2

Values are presented as median (range) and n (%).

CRT = cardiac resynchronization therapy; ICD = implantable cardioverter-defibrillator.

∗ The level of significance was defined as *P* < .05.

### Outcomes

#### Overall visits (in-person visits or virtual visits)

The secular trend established before the pandemic in the non-RM group was one of a gradual decline in overall visits by −5.17 visits per 1000 patients per month (95% CI, −7.00 to −3.34) (Table 2) (Figure 1). In March 2020, there was an immediate further decline in visit volume (level change), after which the trend reversed, increasing by 4.98 visits per 1000 patients per month (95% CI, 1.61–8.35) (Table 2, Figure 1).

**Table 2:** Trend estimates (95% CIs) from best-fitting models of the total number of clinic visits (in person, virtual consult, or both), ED visits, CV hospitalizations, and all-cause mortality among ICD patients with or without RM (per 1000 patients)

Outcome	Monitoring group	Model	Change point (date)	Prechange point trend (95% CI)	Step at change point (95% CI)∗	Postchange point trend (95% CI)
Overall clinic visits	Nonremote	ITS	March 2020	−5.17 (−7.00 to −3.34)	−287.66 (−386.93 to −188.39)	4.98 (1.61–8.35)
	Remote	ITS	March 2020	−3.98 (−5.80 to −2.51)	−44.41 (−143.15 to −2.15)	−0.72 (−4.07 to 2.63)
In-clinic visits	Nonremote	ITS	March 2020	−3.76 (−5.34 to −2.18)	−247.14 (−332.63 to −161.66)	3.55 (0.65–6.45)
	Remote	ITS	March 2020	−1.71 (−2.67 to −0.75)	−172.81 (−224.90 to −120.72)	2.85 (1.08–4.62)
Virtual consults	Nonremote	ITS	March 2020	−1.41 (−2.33 to −0.49)	−40.53 (−90.40 to 9.35)	1.42 (−0.27 to 3.12)
	Remote	ITS	March 2020	−.2.27 (−3.88 to −0.65)	128.39 (40.92–215.86)	−1.56 (−2.00 to −0.60)
ED visits	Nonremote	Linear	N/A	−0.05 (−0.07 to −0.02)	N/A	N/A
	Remote	Segmented	June 2021	−0.02 (−0.03 to −0.01)	N/A	0.35 (−0.16 to 0.87)
CV hospitalization	Nonremote	Linear	N/A	−0.05 (−0.09 to −0.01)	N/A	N/A
	Remote	Linear	N/A	−0.03 (−0.05 to −0.01)	N/A	N/A
All-cause mortality	Nonremote	ITS	March 2020	0.03 (−0.07 to 0.14)	5.67 (0.05–11.29)	−0.18 (−0.04 to 0.17)
	Remote	Linear	N/A	0.01 (0.00–0.021)	N/A	N/A

CI = confidence interval; CV = cardiovascular; ED = emergency department; ICD = implantable cardioverter-defibrillator; ITS = interrupted time series; N/A = not applicable (given that simple linear regression does not have change points); RM = remote monitoring.

∗ A step change is the change in the intercept in March 2020. Step changes only apply to ITS models.

**Figure 1:**
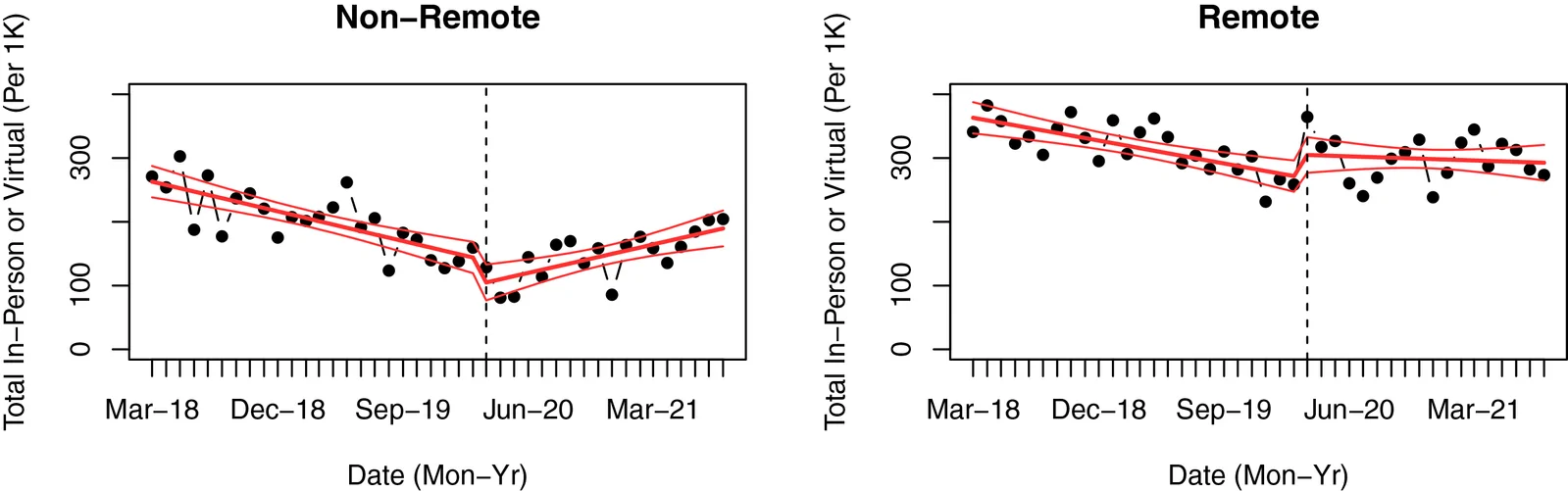
Best-fitting models (*red line*) for the total number of overall visits (per 1000 patients) in both the nonremote and remote groups (*black dots*) by month and year. The *vertical dashed line* indicates the beginning of the pandemic period, March 17, 2020. Mon = month; Yr = year.

In the RM group, there was also a prepandemic secular trend toward fewer visits by a decline of −3.98 visits per 1000 patients per month (95% CI, −5.80 to −2.51) (Table 2). However, in March 2020, the overall visits increased abruptly at the start of the pandemic and then stabilized relative to the prepandemic secular trend with a modest trend toward a decreased number during the intrapandemic period at 0.72 visits per 1000 patients per month (95% CI, −4.07 to 2.63) (Table 2, Figure 1).

#### In-clinic visits

We observed that the trend in in-clinic visits for non-RM patients was toward a decrease before the pandemic, declining by −3.76 visits per 1000 patients per month (95% CI, −5.34 to −2.18) (Table 2), followed by an immediate dip in the level in March 2020. In contrast, the intrapandemic trend was toward an increased number of in-clinic visits, 3.55 visits per 1000 patients per month (95% CI, 0.65–6.45) (Table 2, Figure 2).

**Figure 2:**
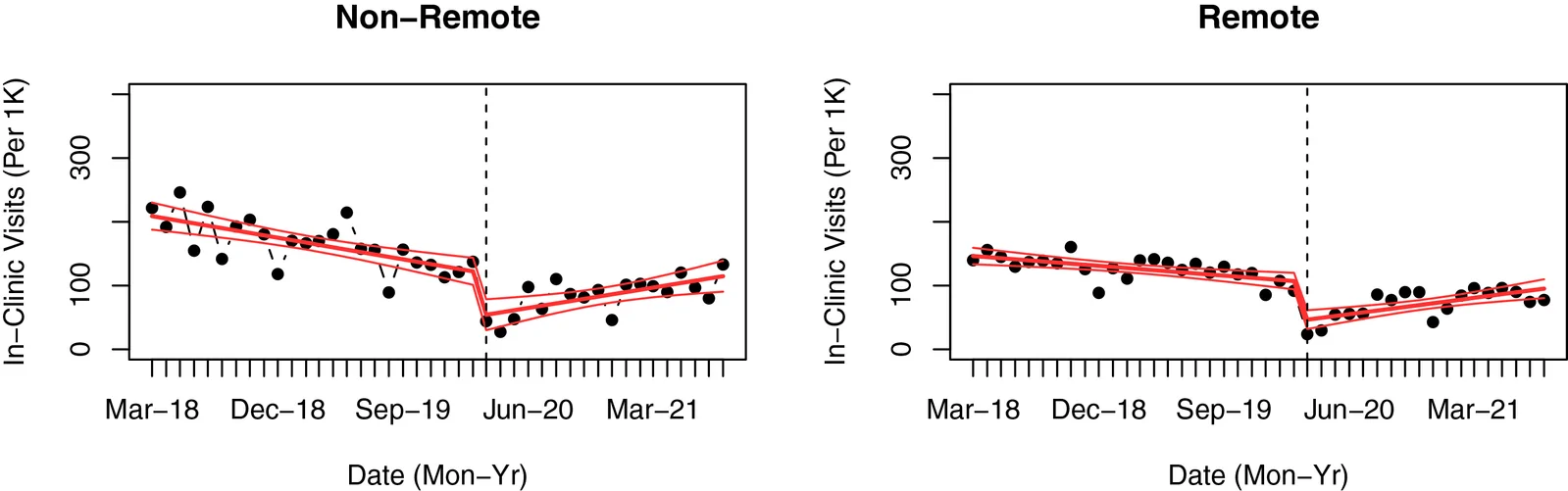
Best-fitting models (*red line*) for the total number of in-person visits (per 1000 patients) in both the nonremote and remote groups (*black dots*) by month and year. The *vertical dashed line* indicates the beginning of the pandemic period, March 17, 2020. Mon = month; Yr = year.

Similarly, the RM group demonstrated a prepandemic secular trend toward fewer in-clinic visits at decreasing −1.17 visits per 1000 patients (95% CI, −2.67 to −0.75) (Table 2). There was a sudden drop in the number of visits at the start of the pandemic in March 2020, which was followed by a marked change in the secular trend toward an increased number of visits with 2.85 visits per 1000 patients per month (95% CI, 1.08–4.62) (Table 2, Figure 2).

#### Virtual consultations

There was a marked difference in the baseline number of visits between non-RM and RM cohorts given that RM patients had approximately 3-fold more baseline visits than non-RM patients at the start of the prepandemic period in 2018.

Among non-RM patients, virtual consultation before the pandemic showed a secular trend toward fewer visits, declining by −1.41 consults per 1000 patients per month (95% CI, −2.33 to −0.49) (Table 2). After a small, immediate increase in level in March 2020, the prepandemic secular trend was modestly reversed as visits increased by 1.42 per 1000 patients per month (95% CI, −0.27 to 3.12) (Table 2, Figure 3). However, the level of increase remained well below the visit numbers of the RM group (Figure 3).

**Figure 3:**
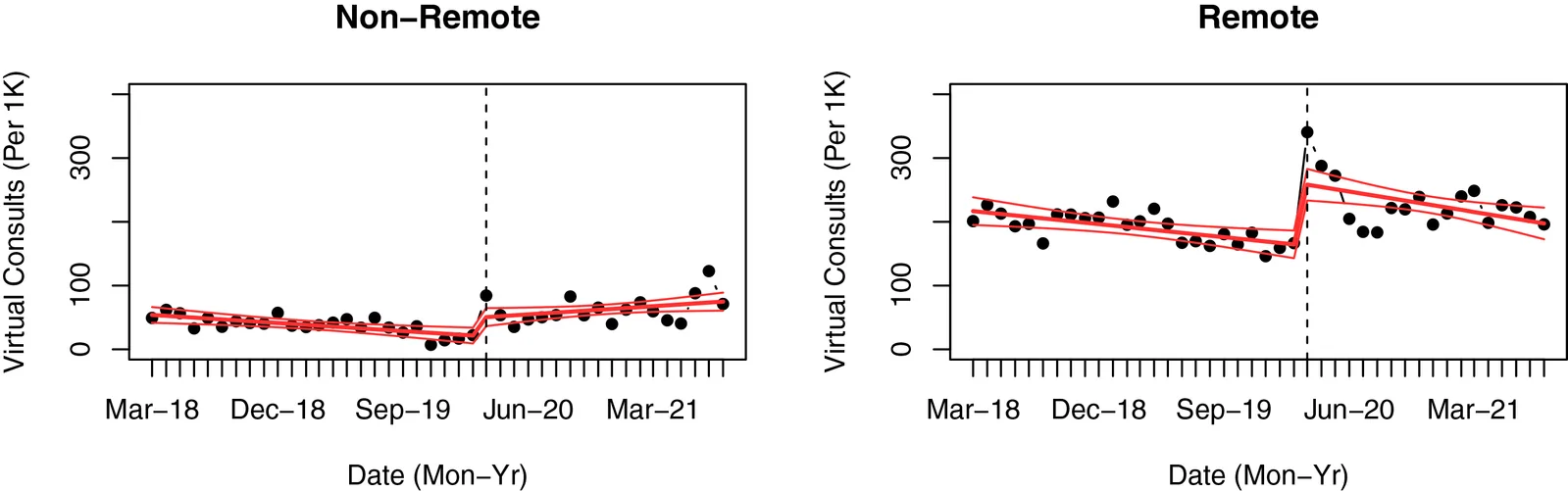
Best-fitting models (*red line*) for the total number of virtual consultations (per 1000 patients) in both the nonremote and remote groups (*black dots*) by month and year. The *vertical dashed line* indicates the beginning of the pandemic period, March 17, 2020. Mon = month; Yr = year.

RM patients, starting from a much higher baseline, demonstrated a downward trend for virtual consultations at a rate of −2.27 consults per 1000 patients per month (95% CI, −3.88 to −0.65) (Table 2) in the prepandemic period. The number of visits immediately increased in March 2020, then declined gradually at a rate of 1.56 consults per 1000 patients per month (95% CI, −2.00 to −0.60) (Table 2, Figure 3).

#### ED visits

Overall, in both the non-RM and RM cohorts, the pandemic did not affect the secular trends, which showed only marginal declines in visits for both cohorts with the non-RM group starting at a higher baseline number of visits.

In the non-RM group, the onset of the pandemic had no effect on ED visits, which continued decreasing at a rate of −0.05 visits per 1000 patients per month (95% CI, −0.07 to −0.02) (Table 2) throughout the study period (Figure 4).

**Figure 4:**
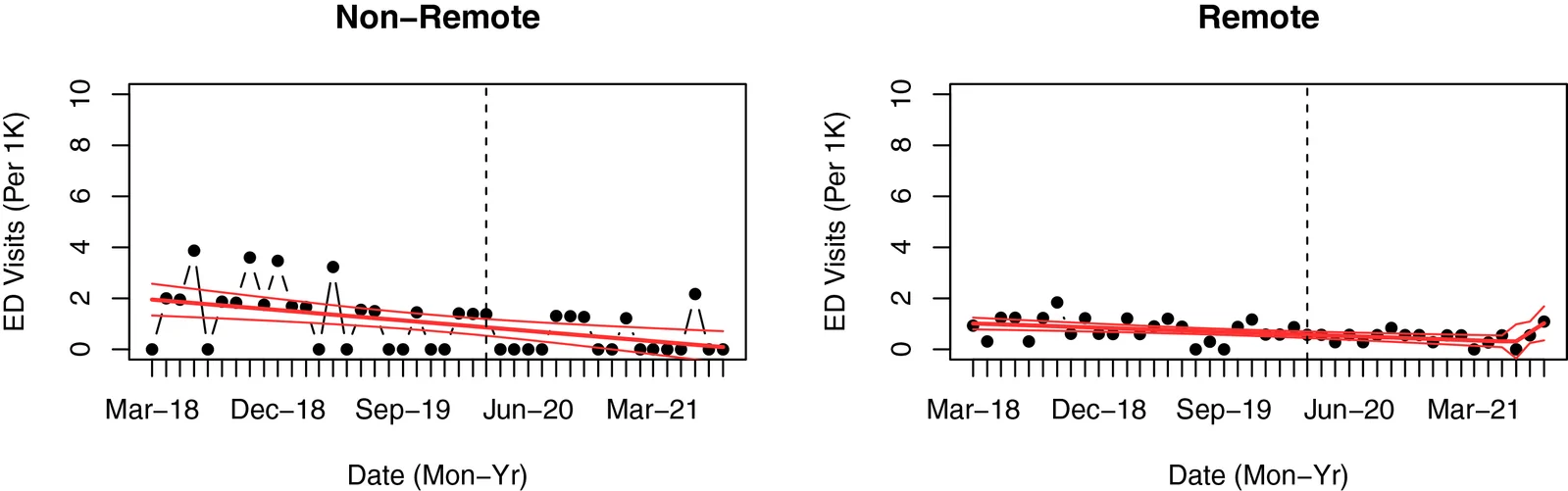
Best-fitting models (*red line*) for the total number of ED visits (per 1000 patients) in both the nonremote and remote groups (*black dots*) by month and year. The *vertical dashed line* indicates the beginning of the pandemic period, March 17, 2020. ED = emergency department; Mon = month; Yr = year.

For the RM group, the trend in the ED visits remained decreasing at a rate of 0.35 per 1000 patient visits per month (95% CI, −0.03 to −0.01) (Table 2) until May 2021 when the trend shifted upward with a rate of 0.35 visits per month, well past the onset of the pandemic (95% CI, −0.16 to 0.87) (Table 2, Figure 4).

#### CV hospitalizations

The trend with respect to CV hospitalizations was minimally downward throughout the study period in both cohorts: non-RM (β1 = −0.05; 95% CI, −0.09 to −0.01) (Table 2) and RM groups (β1 = −0.03; 95% CI, −0.05 to −0.01) (Table 2, Figure 5).

**Figure 5:**
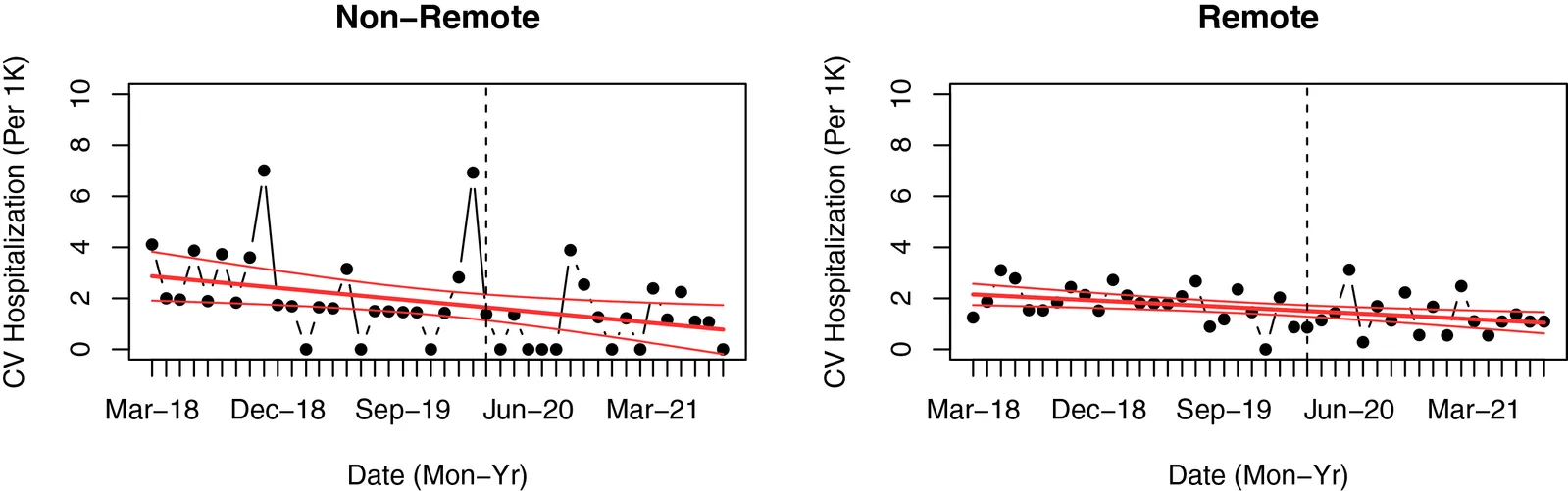
Best-fitting models (*red line*) for the total number of CV hospitalizations (per 1000 patients) in both the nonremote and remote groups (*black dots*) by month and year. The *vertical dashed line* indicates the beginning of the pandemic period, March 17, 2020. CV = cardiovascular; Mon = month; Yr = year.

#### All-cause mortality

Overall, at the beginning of the study period, there was an approximately 2-fold increased rate of mortality in the non-RM group compared with the RM cohort.

In the non-RM cohort, all-cause mortality demonstrated a minimally upward trend before the onset of the pandemic at a rate of 0.03 deaths per 1000 patients per month with wide variance (β1 = 0.03; 95% CI, −0.07 to 0.14) (Table 2). There was a minimal further increase in mortality at the start of the pandemic. However, the mortality secular trend reversed during the intrapandemic period with a decline in the monthly mortality rate of −0.18 deaths per 1000 patients per month (95% CI, −0.04 to 0.17) (Table 2, Figure 6).

**Figure 6:**
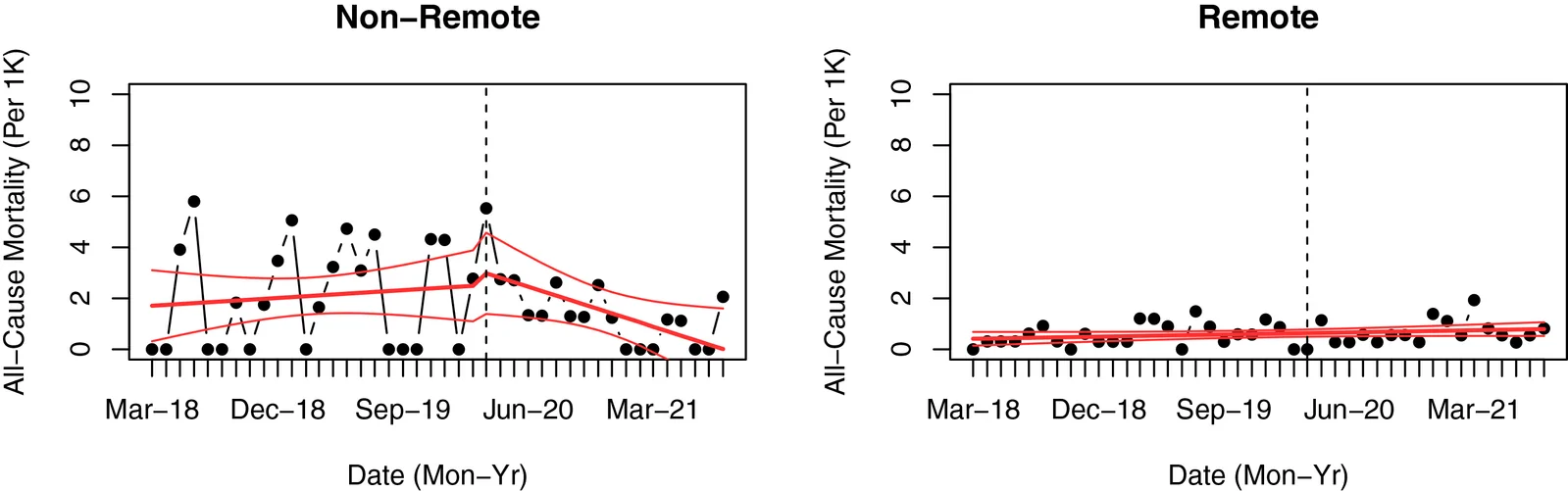
Best-fitting models (*red line*) for the total number of all-cause mortality (per 1000 patients) in both the nonremote and remote groups (*black dots*) by month and year. The *vertical dashed line* indicates the beginning of the pandemic period, March 17, 2020. Mon = month; Yr = year.

In contrast, we found no pandemic effect on the already comparatively low all-cause mortality in the RM group. The established secular trend started at a low baseline and remained essentially unchanged throughout the study period with a slight increased rate of 0.01 per month (95% CI 0.00–0.021) (Table 2) throughout the study period (Figure 6).

## Discussion

To the best of our knowledge, this is the first study to report trends for health utilization and clinical outcomes in defibrillator patients with and without RM during the early months of the COVID-19 pandemic. In this ITS that spanned 3 years before the pandemic and 16 months during the pandemic, we observed some important differences in outcome trends for patients with defibrillators between those with and without RM.

Although both non-RM and RM cohorts had a prepandemic secular trend of a decline in the number of total visits, the RM cohort had a significantly higher baseline rate of visits (Figure 1). This suggests that, during the prepandemic time frame, the RM cohort had overall increased access to clinic services compared with the non-RM cohort (Figure 1). This increased access may be a function of the RM itself whereby RM alerts initiate automatic contact with the clinic. Furthermore, having RM allows patients to have their device interrogated if they develop any symptoms of concern at home, which likely leads to more interactions with the device clinic. In contrast, most interactions between the patient and the device clinic in the non-RM group are patient initiated except for prebooked routine follow-up visits.

At the onset of the pandemic, we observed a sudden sharp decrease in total visits in the non-RM group, followed by a reversal of the secular trend toward an increased rate of visits that remained at an approximately 50% lower rate than the RM group (Figure 1). In contrast, at the onset of the pandemic, there was a sharp increase in the rate of total visits for the RM group (Figure 1). The latter observation strongly suggests that the RM group had better immediate access to clinic services despite the severe constraints related to lack of in-person clinic availability during the first several weeks of the pandemic (Figures 1 and 2).

The increase in overall visits in the RM group is driven by the sharp increase in virtual consultations during the earliest weeks of the pandemic (Figure 3). Although initial prepandemic secular trends were similar, the magnitude of the pandemic effect on the increased rate of virtual consultations in the RM group was much higher than that observed in the non-RM group. The intrapandemic trend for the RM group was a gradual decrease toward the prepandemic baseline virtual visit rate. In contrast, the non-RM group showed only a small increase in virtual consultations, followed by a gradual trend toward an increased rate of visits that remained far below the RM group (Figure 3). This reversal of the secular trend in visit rate during the pandemic suggested that CIED clinics improved virtual access for both groups during the pandemic, but that increased clinic access remained much greater for the RM group (Figures 1 and 3).

The finding of improved immediate access to care for the RM group at the onset of the pandemic has several possible explanations. First, RM patients had a preexisting digital infrastructure, which enabled a rapid pivot to virtual care. RM patients already had established electronic communication pathways (device-to-server data transmission, nurse-initiated callbacks). When in-person visits were curtailed, these pathways were immediately convertible to virtual consultations without requiring new enrollment, equipment, or workflow redesign. This would potentially facilitate earlier access to care in the event of a sudden disruption of in-person access that occurred during the early days of the pandemic. Second, RM may generate alerts related to arrhythmias, lead integrity issues, battery depletion, and heart failure diagnostics. These alerts may trigger nurse clinician contact regardless of clinic access constraints. In contrast, non-RM patient contact with the clinic is dependent on patient-initiated contact outside regularly scheduled visits.

For in-person visits, although the RM group started at a higher baseline rate of visits, there was a near-identical secular trend slope, pandemic effect, and intrapandemic effect that led to an abrupt early pandemic decrease in visits, followed by a later gradual increase in the in-person visits for both groups. This observation suggests that the pandemic had a nearly identical effect on the secular trends in both groups for in-person visits. This finding suggests that government-mandated and institutional pandemic-related restrictions on in-person clinic visits were applied with a similar effect on both groups.

The residual plots showed no clear pattern or strong correlation over time, suggesting that any serial correlation present is minimal and unlikely to affect the model results (Supplemental Table 2, Supplemental Figures 1 and 2).

For the ED visits outcome, there was a minimal pandemic effect on the secular trend. However, a modest upward trend was observed in ED visits for the RM group toward the end of the pandemic study period. This finding may be explained by a decline in pandemic-related fear to seek emergency care or may have reflected a function of health system reopening (Figure 4). In addition, some clinics, in the face of restricted access to in-person device clinic care, may have advised RM patients to visit the nearest ED outside of clinic hours or on weekends.

Previous studies have reported that RM has been associated with a lower mortality rate than no-RM management.^14^, ^15^, ^16^ The non-RM group demonstrated an increased baseline mortality trend before the pandemic compared with the RM group but shifted modestly toward a lower rate during the pandemic. These findings are likely explained by the higher risk population characteristics of the non-RM group including higher numbers of resynchronization defibrillator patients and patients with heart failure, atrial fibrillation, and diabetes at baseline. Compared with the non-RM cohort, the RM group had a lower baseline mortality rate, which had a minimal downward trend in mortality rate but no observable pandemic or intrapandemic effect. Thus, it is likely that the pandemic had a small effect on mortality in the non-RM group, which is not easily explainable, although there was an observed increase in virtual visits during the pandemic. It remains possible that the observed trend of decreased mortality in the non-RM cohort is a statistical regression to the mean or because of a survival bias effect whereby people with the highest level of comorbidity in this group died early and those who survived had fewer comorbidities and hence had a trend toward a lower mortality rate during the study period.

Other studies have recommended that governments should consider the adoption of RM to ensure access is always optimized for defibrillator patients.^15^^,^^17^ Furthermore, RM supports chronic disease management, reduces hospitalizations, and enhances access in underserved areas. Given that previous studies confirm RM’s cost-effectiveness, both public and private health purchasing agencies should consider expanded adoption of RM.^15^^,^^17^ Our study findings support these recommendations especially regarding RM patients’ access to care during periods of constrained in-person CIED clinic availability.

Our study has limitations. First, we did not control for potential confounding factors, which could have influenced our results. The use of propensity score matching, based on baseline characteristics, could have provided potentially more accurate and adjusted outcomes, but matching would have reduced the sample size in our study. Second, because of the ITS design, causality for changes in secular trends is not definitive owing to unmeasured patient characteristics. Our data only establish association and not causality about the pandemic and outcomes measured in our study. Third, inclusion of data on primary vs secondary defibrillator implant indication and associated left ventricular ejection may have enhanced our ability to detect differences between RM and non-RM in these subgroups. However, these data were not directly available to us. The large, representative and pragmatic sample of ICD patients in our study allowed us to detect subtle trends that contributed to the robustness of our results.

Thus, the summary of our findings is that the RM group had a higher baseline virtual visit rate that was magnified by the pandemic. In-person visit rate trends were similar in both RM and non-RM groups, and these trends were affected by the pandemic. Furthermore, there was a near-identical trend of decrease in in-person visits at the onset of the pandemic that led to a surge in virtual consultations that was of markedly higher magnitude in the RM group. Finally, despite evidence of increased access to clinic care overall in the RM group, there was no significant pandemic effect on mortality, CV hospitalizations, or emergency room visits.

## Conclusion

Compared with a non-RM cohort of defibrillator patients, the RM cohort maintained a higher level of CIED clinic access at the onset of the COVID-19 pandemic, which was driven by improved access to virtual device clinic care. Despite this improved access to care, secular trends in CV hospitalizations, ED visits, and mortality remained minimally affected by the pandemic.

## Disclosures

The authors have no conflicts of interest to disclose.

## Appendix Supplementary Data

Supplementary Figure 2

Supplementary Equation and Supplementary Tables

## Footnotes

Appendix Supplementary data associated with this article can be found in the online version at https://doi.org/10.1016/j.hroo.2026.06.008.

## References

[1] Urrutia D., Manetti E., Williamson M., Lequy E. (2021). Overview of Canada’s answer to the COVID-19 pandemic’s first wave (January-April 2020). Int J Environ Res Public Health.

[2] Chew D.S., Zarrabi M., You I. (2022). Clinical and economic outcomes associated with remote monitoring for cardiac implantable electronic devices: A population-based analysis. Can J Cardiol.

[3] Varma N., Epstein A.E., Irimpen A., Schweikert R., Love C., TRUST Investigators (2010). Efficacy and safety of automatic remote monitoring for implantable cardioverter-defibrillator follow-up: the Lumos-T safely reduces routine office device follow-up (TRUST) trial. Circulation.

[4] Ricci R.P., Vicentini A., D’Onofrio A. (2013). Impact of in-clinic follow-up visits in patients with implantable cardioverter defibrillators: demographic and socioeconomic analysis of the TARIFF study population. J Interv Card Electrophysiol.

[5] Crossley G.H., Boyle A., Vitense H., Chang Y., Mead R.H., CONNECT Investigators (2011). The CONNECT (clinical evaluation of remote notification to reduce time to clinical decision) trial. J Am Coll Cardiol.

[6] Ferrick A.M., Raj S.R., Deneke T. (2023). HRS/EHRA/APHRS/LAHRS expert consensus statement on practical management of the remote device clinic. Europace.

[7] Varma N., Marrouche N.F., Aguinaga L. (2021). HRS/EHRA/APHRS/LAHRS/ACC/AHA worldwide practice update for telehealth and arrhythmia monitoring during and after a pandemic. Europace.

[8] Lakkireddy D.R., Chung M.K., Gopinathannair R. (2020). Guidance for cardiac electrophysiology during the COVID-19 pandemic from the Heart Rhythm Society COVID-19 Task Force; Electrophysiology Section of the American College of Cardiology; and the Electrocardiography and Arrhythmias Committee of the Council on Clinical Cardiology, American Heart Association. Heart Rhythm.

[9] ICD-10 Version: 2019 International Statistical Classification of Diseases and Related Health Problems 10th Revision. https://icd.who.int/browse10/2019/en.

[10] Loitz C.C., Johansen S., Johnston J.C. (2023). Exploring the Alberta Health Services COVID-19 provincial public health integrated outbreak prevention, preparedness, management, and response interventions to support congregate and communal living sites in Alberta. Can J Public Health.

[11] Yee R., Verma A., Beardsall M., Fraser J., Philippon F., Exner D.V. (2013). Canadian Cardiovascular Society/Canadian Heart Rhythm Society Joint Position Statement on the Use of Remote Monitoring for Cardiovascular Implantable Electronic Device Follow-up. Can J Cardiol.

[12] Kelly S.E., Campbell D., Duhn L.J. (2021). Remote monitoring of cardiovascular implantable electronic devices in Canada: survey of patients and device health care professionals. CJC Open.

[13] Medtronic Cardiac rhythm & diagnostics. https://www.medtronic.com/en-ca/healthcare-professionals/products/cardiac-rhythm.html.

[14] Iqbal F.M., Lam K., Joshi M., Khan S., Ashrafian H., Darzi A. (2021). Clinical outcomes of digital sensor alerting systems in remote monitoring: a systematic review and meta-analysis. NPJ Digit Med.

[15] Chew D.S., Piccini J.P., Frazier-Mills C., Michalski J., Varma N. (2024). Alert-based remote monitoring: A model for increased reimbursement to meet device clinic workload while achieving overall health system cost savings. Heart Rhythm.

[16] Varma N., Piccini J.P., Snell J., Fischer A., Dalal N., Mittal S. (2015). The relationship between level of adherence to automatic wireless remote monitoring and survival in pacemaker and defibrillator patients. J Am Coll Cardiol.

[17] Nishii N. (2014). Remote monitoring of cardiac implantable electronic devices. J Arrhythm.

