# Supplementary material for: The impact of remote monitoring on clinical outcomes in defibrillator patients during the COVID-19 pandemic: An interrupted time series analysis
Source: Heart Rhythm O2. 2026 Jun 19;7(10):1971–8. doi: 10.1016/j.hroo.2026.06.008 (PMC13700346; doi:10.1016/j.hroo.2026.06.008)
Supplement: Supplementary file 1 [file mmc1.pdf]

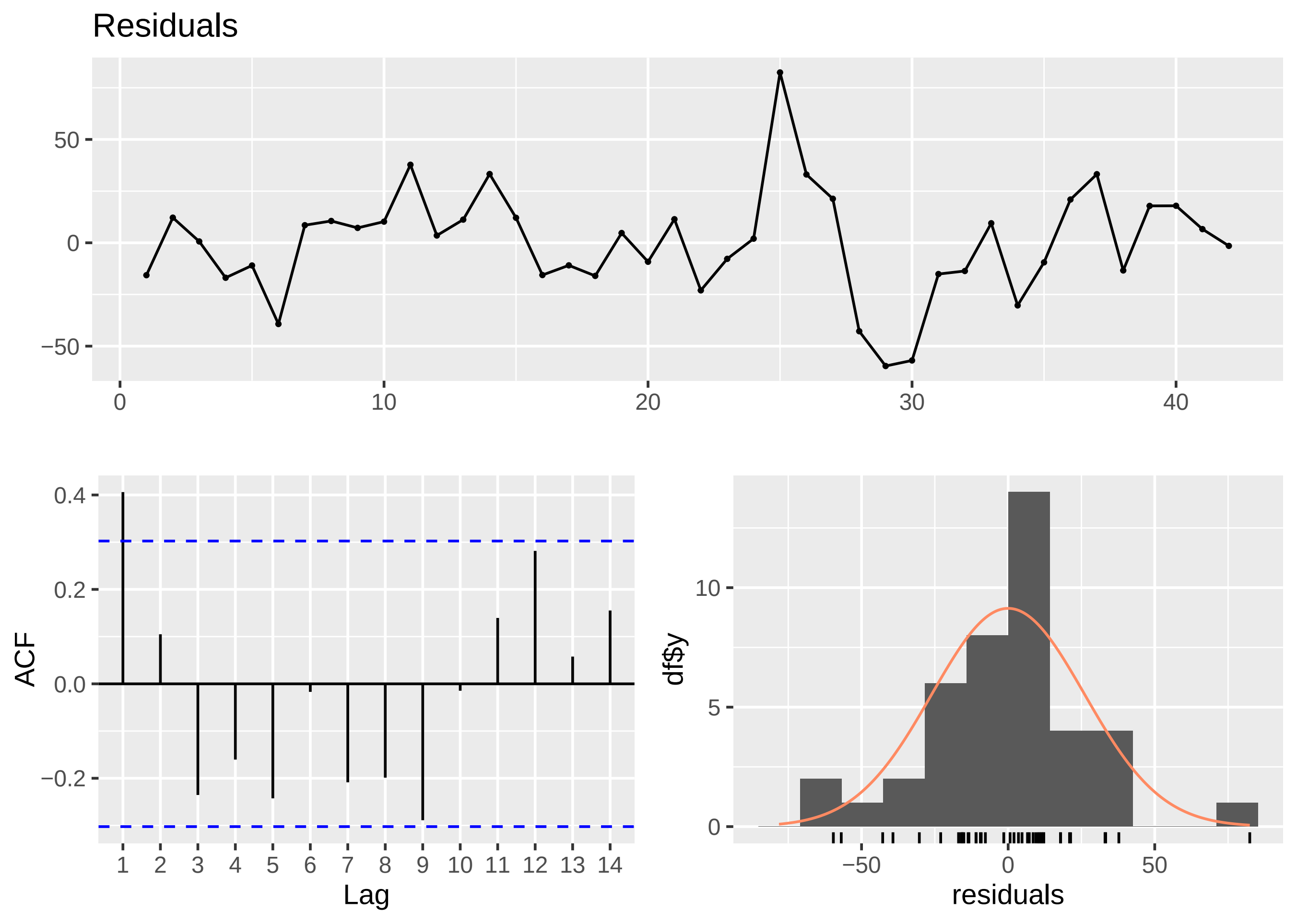

Residuals
50
0
−50
0
10
20
30
40
ACF
0.4
0.2
0.0
−0.2
1
2
3
4
5
6
7
8
9
10
11
12
13
14
Lag
df$y
10
5
0
−50
0
50
residuals
