# Supplementary material for: The impact of remote monitoring on clinical outcomes in defibrillator patients during the COVID-19 pandemic: An interrupted time series analysis
Source: Heart Rhythm O2. 2026 Jun 19;7(10):1971–8. doi: 10.1016/j.hroo.2026.06.008 (PMC13700346; doi:10.1016/j.hroo.2026.06.008)
Supplement: Supplementary file 3 [file mmc3.docx]

**SUPPLEMENTARY**

**EQUATION**

**Supplementary Equations:** The three different regression models are: linear regression model, interrupted time series model, and segmented regression model:

1. a standard linear regression model with monthly time points as the sole independent variable, $\hat{y}=\hat{\beta_{0}}+\hat{\beta_{1}}time$;

2.	an interrupted time series (ITS) model with March 2020 included as a “dummy” variable to represent healthcare responses to COVD-19, $\hat{y}=\hat{\beta_{0}}+\hat{\beta_{1}}time+\hat{\beta_{2}}(pandemic)+\hat{\beta_{2}}(pandemic\times time)$; and

3.	a segmented regression model, where an outcome is modelled as two separate linear functions on either side of a breakpoint (or “hinge”, *h*). Here, h was automatically chosen by using the R package *segmented* (version 1.6-4) to produce the fitted model, $\hat{y}=\hat{\beta_{0}}+\hat{\beta_{1}}time+\hat{\beta_{2}}(time-h)I_{h}$, where $I_{h}=0$ when $time\leq h$ and $I_{h}=1$ when $time>h$.

For reach model, $\beta_{0}$ represents the baseline level at time = 0, $\beta_{1}$ is the slope of an outcome per month (representing the underlying trend before March 2020); in the ITS model, $\beta_{2}$ is a level change following March 2020, and $\beta_{3}$ indicates the slope change following March 2020; $\beta_{2}$ in the segmented model indicates the slope following a hinge point, h.

**TABLES**

**Supplementary Table 1:** International Classification of Diseases, Tenth Revision codes for identification of comorbidities

| **Comorbidities** | **ICD-10 codes** |
| --- | --- |
| Arrhythmia | I46*, I47*, I48*, I49.0*, I49.8, I49.9 |
| Atrial fibrillation/atrial flutter | I48* |
| Coronary or peripheral vascular disease | I20.0, I20.1, I208.0, I208.8, I20.9, I21*, I22*, I23*, I25.2, I70.2*, I70.8, I70.9, I73.8, I73.9 |
| Diabetes | E10*, E11*, E12*, E13*, E14* |
| Dyslipidemia | E78 |
| Heart failure | I50.0, I50.1, I50.9 |
| Hypertension | I10*, I11, I12, I13, I15*, I27* |
| Ischemic Cardiomyopathy | I255 |
| Non-ischemic Cardiomyopathy | I42 |
| Stroke | I63*, I69.3, I64 |
| Supraventricular tachycardia | I47.1 |
| Ventricular fibrillation/flutter | I47.2 |
| Sudden cardiac arrest | I46* |
| myocardial infarction | I21, I22 |
| Renal disease | N18*, N19 |

**Supplementary Table 2.** Breusch-Godfrey test for serial correlation in the residuals (up to order 8) for each model presented in the main text. P-values < 0.05 suggest evidence for serial correlation.

| **Outcome** | **Monitoring Group** | **Model** | **Breusch-Godfrey**  $\boldsymbol{\chi}^{\boldsymbol{2}}$ **(df)** | ***p*** |
| --- | --- | --- | --- | --- |
| Overall Clinic Visits | Non-Remote | ITS | 7.47 (8) | 0.486 |
|  | Remote | ITS | 11.83 (8) | 0.159 |
|  |  |  |  |  |
| In-Person Visits | Non-Remote | ITS | 7.16 (8) | 0.519 |
|  | Remote | ITS | 10.47 (8) | 0.234 |
|  |  |  |  |  |
| Virtual Consults | Non-Remote | ITS | 15.83 (8) | **0.045** † |
|  | Remote | ITS | 17.82 (8) | **0.023** † |
|  |  |  |  |  |
| ED Visits | Non-Remote | Linear | 5.55 (8) | 0.698 |
|  | Remote | Linear | 10.37 (8) | 0.240 |
|  |  |  |  |  |
| CV Hospitalization | Non-Remote | Linear | 5.53 (8) | 0.698 |
|  | Remote | Segmented | 9.28 (8) | 0.319 |
|  |  |  |  |  |
| All-Cause Mortality | Non-Remote | ITS | 12.25 (8) | 0.140 |
|  | Remote | Linear | 9.46 (8) | 0.305 |
|  |  |  |  |  |

**Abbreviations:** CI = Confidence interval; CV = Cardiovascular; ED = Emergency department;

ITS = Interrupted time series; N/A = Not applicable, since simple linear regression does not have change points.

**Serial Correlation:** Serial correlation (or autocorrelation) in a time series regression model describes a relationship where the model's residuals at month t are correlated with the residuals at previous (t - k) months (where k is referred to as the lag). It implies that data are not independent, where past observations can predict future ones. If ignored, serial correlation violates the assumptions of ordinary least squares, leading to inefficient estimates, incorrect standard errors, and misleading or even false conclusions about statistical significance.

† = residual plots contradict the p-value of the Breusch-Godfrey test and do not indicate large amounts of serial correlation.

**FIGURES**

**Supplementary Fig. 1:** Graphical residual diagnostics for ITS models with significant Breusch-Godfrey tests up to 8th order autocorrelation for remote monitoring patients for virtual visits.

Panel shows a time plot (top), the autocorrelation function (ACF, bottom left), and the histogram of the residuals (bottom right).

- The time plot shows that the changing variation over time and is unremarkable.

- The autocorrelation plot illustrates a significant spike at lag 1 (i.e., the correlation with the previous month exceeds the blue dashed line), but it is not particularly large, and would be unlikely to have any noticeable impact on the forecasts or the prediction intervals-which are not performed in this paper.

- The histogram illustrates that the residuals have slight positive kurtosis.

**Supplementary Fig. 2:** Graphical residual diagnostics for ITS models with significant Breusch-Godfrey tests up to 8th order autocorrelation for non-remote monitoring patients for virtual visits. Panel shows a time plot (top), the autocorrelation function (ACF, bottom left), and the histogram of the residuals (bottom right).

-The time plot illustrates that the changing variation over time and is unremarkable.

-The autocorrelation plot did not illustrate any significant spike (i.e., the correlation with the -previous month exceeds the blue dashed line)

-The histogram illustrates that the residuals have slight positive kurtosis.
